# An Overview of Reviews on Predictors of Neurorehabilitation in Surgical or Non-Surgical Patients with Brain Tumours

**DOI:** 10.3390/life14111377

**Published:** 2024-10-26

**Authors:** Mattia Gambarin, Tullio Malgrati, Rita Di Censo, Angela Modenese, Giulio Balestro, Gloria Muti, Marta Cappellesso, Cristina Fonte, Valentina Varalta, Ylenia Gallinaro, Monica Pinto, Matilde Carlucci, Alessandro Picelli, Nicola Smania

**Affiliations:** 1 Neurorehabilitation Unit, Department of Neurosciences, Hospital Trust of Verona, 37126 Verona, Italy; 2 Neuromotor and Cognitive Rehabilitation Research Centre (CRRNC), Department of Neurosciences, Biomedicine and Movement Sciences, University of Verona, 37129 Verona, Italy; 3 National Cancer Institute Pascale Foundation IRCSS, 80131 Napoli, Italy; 4 Healthcare Directorate, Hospital Trust of Verona, 37126 Verona, Italy

**Keywords:** rehabilitation outcomes, brain tumour, quality of life

## Abstract

(1) Background. People suffering from brain cancer, regardless of histological tumour characteristics, often experience motor disturbances, cognitive–behavioural difficulty, language impairments, and functional and social limitations. The current treatment approach entails surgery and adjuvant therapy such as chemotherapy and radiotherapy combined with intensive rehabilitation. The primary focus of rehabilitation is usually motor and functional recovery, without specifically addressing the patient’s quality of life. The present systematic review identifies and evaluates the predictors of functional and cognitive rehabilitation outcomes and their influence on quality of life in adult patients with brain cancer. (2) Methods. Three electronic databases (PubMed, Elsevier, Cochrane) were searched for reviews about functional, cognitive, and quality-of-life outcomes in patients with central nervous system tumours, including articles published between January 2018 and May 2024. (3) Results. The search retrieved 399 records, 40 of which were reviewed. Five main areas of predictive factors were identified: diagnosis, therapy, complications, outcomes (in the motor, cognitive, and quality-of-life categories), and tailored rehabilitation. (4) Conclusions. These indicators may inform integrated care pathways for patients with primary central nervous system tumours.

## 1. Introduction

Patients with primary central nervous system (pCNS) tumours experience a variety of motor, cognitive, and clinical symptoms that are directly influenced by the type of tumour itself and its location in the brain. Surgery [1], radiotherapy, chemotherapy, and pharmacotherapy can all exacerbate these symptoms [2,3].

The multifactorial pattern of impairment and symptoms often includes motor deficits, cognitive deficits, aphasia, dysphagia, confusion, seizures, drowsiness, headache, fatigue, and dyspnoea. To lengthen survival, patients with aggressive brain tumours often undergo surgical resection, chemotherapy, and radiotherapy, which carry risks of neurological disability and complications that can reduce quality of life and compliance with rehabilitation [1,2]. This means that people with brain tumours constantly face hard choices about their treatment and management options, in which they seek to balance the benefits of treatment against the side effects and weigh survival rates against expected quality of life.

The concept of health-related quality of life (HRQoL) encompasses physical, psychological, and social well-being [4]. It follows, then, that rehabilitation will rely on a holistic approach to meet patient needs. Such an approach will take into account both clinical and rehabilitative outcomes that may or may not affect HRQoL, as well as patient perception of disease and quality of expected survival [4,5]. Currently, there are no robust guidelines for rehabilitation management in patients with brain cancer, and there is little concrete information about the impact of surgical and adjuvant therapies and symptom variability in rehabilitation [6,7].

Multidisciplinary-team-based models of survivorship care are being introduced to better and more quickly address the complex psychological, physical, and health needs of cancer patients. Integrated patient-centred approaches are associated with greater patient satisfaction, quality of care, and quality of life; fewer mistakes; and quicker diagnostic response. Multidisciplinary care management fosters coordination between health care units and allows them to focus on a specific patient group [8].

Physical medicine and rehabilitation specialists are key members of such teams. They help patients to identify functional, cognitive, and psychological factors/outcomes when making therapeutic decisions, adapt rehabilitation to their expectations and needs, and increase patient satisfaction and compliance [2]. However, because robust guidelines on outcomes and patient experience are lacking, rehabilitation programmes often lack specificity [6].

The European Cancer Code of Practice (ECCP) sets out ten basic rights for cancer patients. The ECCP stresses the importance of providing patients with high-quality information about their disease. The ECCP recognises the importance of multidisciplinary cancer care teams, shared decision-making, research, and innovation, with a focus on quality of life and supportive care. It also recommends a systematic approach to supporting cancer survivors with survivorship care plans [9]. Patient satisfaction increases compliance, improves clinical outcomes, and creates retention and referrals [10]. Information about patient needs includes prognosis, medical and clinical complications, treatment side effects, functional and cognitive recovery following invasive treatments, and expected quality of life [2]. Appropriate neuro-oncological rehabilitation programmes should therefore respect the patient rights set out in the ECCP, particularly as regards level of assessment, monitoring, information, support, and multidisciplinary intervention. 

Clinical outcomes, such as survival, motor and cognitive function, and quality of life, usually indicate the success of therapy and rehabilitation, but considering patients’ personal experience and satisfaction as well may help in tailoring the project of care and improving well-being for long-term conditions. Therefore, the present review aims to define rehabilitation outcome criteria and the factors that can influence patient-perceived quality of life. The primary study objective was to identify and evaluate predictors of functional and cognitive rehabilitation outcomes in adult patients with brain cancer and to assess the factors that influence quality of life. A secondary objective was to determine how symptom burden, treatment side effects, and surgical intervention may affect quality of life both subjectively and objectively. The predictors were grouped into five domains: diagnosis, therapy, complications, outcomes (including motor, cognitive, and quality-of-life outcomes), and tailored rehabilitation, and they are discussed according to the ECCP. For the present study, we focused on reviews, systematic reviews, and meta-analyses to have an up-to-date overview of available evidence. This choice enabled us to identify gaps in the literature where further research is needed.

Our data may provide valuable insights for developing personalised rehabilitation programmes for neuro-oncology patients and facilitate the integration of care and rehabilitation in multidisciplinary team management.

## 2. Methods

The online databases PubMed, Cochrane, and Elsevier (Scopus) were queried using the search terms: ((brain neoplasm) OR (meningioma) OR (glioma) OR (glioblastoma)) AND ((rehabilitation) OR (cognitive)) AND ((outcomes) OR (quality of life)).

This review was conducted and reported according to the Preferred Reporting Elements for Systematic Reviews and Meta-Analyses (PRISMA) Guidelines and the Overview of Reviews section. The inclusion criteria were as follows: reviews, systematic reviews, and meta-analyses published in English between January 2018 and April 2024, with a focus on, or at least a discussion of, functional outcomes, cognitive outcomes, or quality of life in adult patients undergoing primary CNS tumour treatment. The studies had to report on predictors of neuromotor and cognitive outcomes and quality of life in neuro-oncology/neurorehabilitation. The reference lists of some studies were manually searched for other articles of interest. The exclusion criteria were as follows: non-neurological malignancies, non-CNS tumours, surgical procedures, terminally ill patients, pharmacological therapy outside the rehabilitation setting, and children. Unpublished papers were also excluded.

Two reviewers independently screened potentially eligible titles and selected studies, extracted data, and assessed study quality; a third investigator was involved in resolving disagreements about whether to include a paper or not. Duplicates were eliminated using the Zotero software package, version 6.0.37, 2024.

## 3. Results

**Figure 1:**
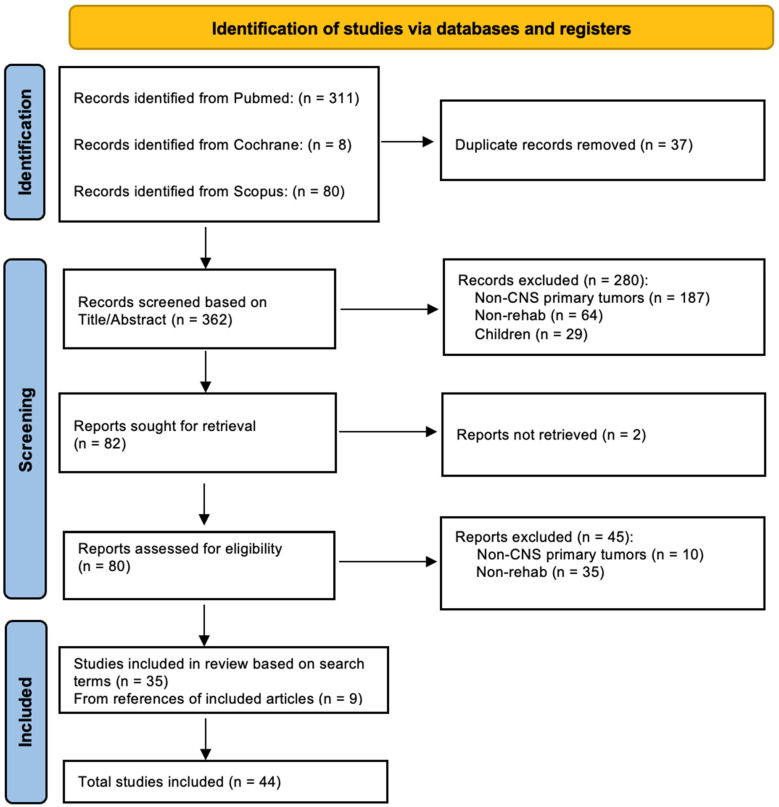
Flowchart for identification of included and excluded studies. A double screening approach was used.

The literature search yielded 399 records, 37 of which were removed because they were duplicates; 280 of the remaining 362 records were excluded because they did not meet the inclusion criteria; 45 of the remaining 82 records were excluded because they did not investigate rehabilitation (n = 35) or primary CNS tumours (n = 10). The remaining thirty-five records were included, and nine more were added from searching the references, for a total of forty-four records eligible for review. Further details are given in Figure 1.

The 44 studies were searched for functional and cognitive outcomes and quality of life. The predictors were grouped into five domains: diagnosis, therapy, complications, outcomes (in the motor, cognitive, and quality-of-life categories), and tailored rehabilitation. The predictors categorised under the Diagnosis domain were patient characteristics, tumour type and location, grade, and genetic information. The predictors under the Therapy domain were surgical resection, chemotherapy, radiotherapy, antiepileptics, and corticosteroids. The predictors under the Complications domain were perilesional oedema, seizures, and fatigue. The predictors under the Outcome Measures domain were cognitive state, motor dysfunction, patient needs, and quality of life. Finally, the predictors under the Tailored Patient Rehabilitation domain were adaptation to disease progression, neuromotor, cognitive, and multidisciplinary team involvement.

### 3.1. Tumour Diagnosis

#### 3.1.1. Patient Characteristics

Specific characteristics of patients with grade II glioma, such as older age (>40 years), indicated poorer prognosis [11]. High education level appeared to indicate better neurocognitive function (NCF), while older age, carrying the APOE e4 allele, and other germline genetic polymorphisms were associated with increased risk of NCF dysfunction [12]. Personality, affective, or neurobehavioural changes—such as depression or anxiety, sleep disturbances, and fatigue—may be noted.

In patients with low-grade glioma, reduced affective expression was often observed, which correlated with lower executive function performance. Depression and executive dysfunction were associated with survival, further exacerbated by affective distress and neurocognitive deficits [12].

Patient age and Karnofsky Performance Scale (KPS) score, together with tumour site and genetic and molecular characteristics (see below), are determinants of functional impairment and prognosis recognised by the World Health Organisation (WHO) and outlined in the European Association of Neuro-Oncology (EANO) guidelines published in 2021 by Weller et al. [13,14]. The ϵ4 allele of apolipoprotein E (ApoE) is a genetic variant critical for neuronal growth and repair and associated with increased susceptibility to cognitive and language dysfunction in such brain tumours as glioblastoma. Patients with this genetic variant may experience difficulty with memory, attention, and other cognitive functions, especially after chemoradiation therapy, which is effective against cancer but can cause cognitive decline as a side effect [15,16].

#### 3.1.2. Tumour Site

We found seven reviews on tumour site and outcomes. Several included data on brain tumour site and clinical outcomes, while others reported data on brain connectivity network involvement and clinical outcomes. Reviews investigating the relationship between clinical presentation and lesion side have shown that left brain tumours, particularly gliomas in the dominant hemisphere (often the left), tend to cause more severe cognitive dysfunction, such as aphasia, verbal learning disorder, and problems with verbal intelligence, than those arising in the right brain [12,15,16].

Frontal lobe tumours alter visuospatial categorisation, visual memory, and digital symbol substitution. High-grade right prefrontal gliomas affect executive and visuospatial functions, resulting in impaired activity initiation, maintenance of mental productivity, inhibitory control, self-monitoring, and response regulation. Non-frontal malignant brain tumours can also affect executive function by putting pressure on the neural networks responsible for these functions. The most commonly affected areas are the default-mode network and the executive control network, both of which are critical for executive function. Frontotemporal tumours affect emotional, recognition, and processing. Tumours in the insula affect affective and cognitive empathy more than other types of glioma. Tumours in the anterior lobe of the cerebellum are associated with altered motor function, while those located in the vermis are associated with emotional processing, and those in the posterior lobe with complex cognitive functions [17].

Studies investigating subcortical connectivity and clinical correlations applied intraoperative mapping and real-time behavioural monitoring in awake patients. The studies compared perioperative neuropsychological and neuroimaging data which revealed the dynamics of intra- and inter-neuronal interaction loops. The concept of a meta-network (network of networks) and the role of subcortical connectivity are viewed as an important constraint on neuroplastic potential. The studies investigating the anatomical location of gliomas and functional neurocognitive neural networks reported that the compression on/involvement of functional neural networks may have a greater impact on symptoms than mere tumour location. This is particularly true for high-flow functional areas (the “hub” centres) for rapid information processing in the brain [17,18,19,20]. As an example of the interaction between tumours and the connectome, gliomas affecting the frontal and the temporal lobes may be correlated with impairments in language, learning ability, memory, and executive functions [18,19]. A very useful concept in rehabilitation is the clinical correlation between eloquent areas and tumour sites. Krishna and colleagues suggested that behavioural disorders and functional recovery difficulties arise when tumours invade “eloquent” brain regions, affecting subcortical pathways or extending to the cortico-subcortical region. This phenomenon is probably due to the limited plasticity of the eloquent cortical areas, owing to the lack of alternative functional circuits, and impairment of the subcortical tracts’ remodelling capacity after a lesion develops [16]. Duffau et al. extended the concept of subcortical pathways to the eloquent language area; they pointed out that the language circuit is not a single entity but rather comprises various subcircuits (including dorsal and ventral streams) that interact with other networks, particularly those that support executive functions and attentional processing, which suggests the utility of therapeutic interventions designed to strengthen broken connections. The hypothesis is that interventions combining functional/cognitive rehabilitation with transcranial brain stimulation may strengthen/restore damaged meta-network connections [20].

#### 3.1.3. Tumour Genetics

Different types of tumours may affect the functional network to various extent. IDH1-WT gliomas, which grow faster, worsen neurocognitive function compared with IDH1-Mut gliomas, despite similar tumour size. Kesler’s studies in Cooman et al.’s review on IDH1-mutant and IDH1-WT malignant astrocytomas showed that IDH-WT gliomas are associated with a higher cognitive load than IDH-Mut tumours, and that the brain connectivity of IDH1-WT tumours is lower than that of IDH1-mutant gliomas, particularly in medial frontal, posterior parietal, and subcortical sites [17,19,21,22,23]. Patients with higher-grade tumours or/and IDH-WT gliomas exhibit worse NCF than patients with lower-grade or IDH-Mut tumours; those with IDH1-WT gliomas perform worse than those with IDH-Mut gliomas on learning, memory, processing speed, language, executive function, and dexterity tests [24]. IDH-mutated tumours have a relatively low cognitive load because they grow slower. This causes less damage to neural networks and preserves cognitive function [12,24]. The association of IDH status with quality of life remains less clear [25].

#### 3.1.4. Tumour Grade

Tumour grade was directly correlated with a range of disabilities, including cognitive, motor, and behavioural difficulties. In gliomas, this is due to the impact of tumour growth kinetics on the brain’s neuroplasticity and the organisational response within the cerebral connective network. It has been observed that high-grade tumours can infiltrate and migrate rapidly, exceeding the capacity of natural remodelling. In contrast, low-grade gliomas progress more slowly, thus allowing sufficient time for functional network reorganisation to occur [16]. In high-grade brain tumours such as glioblastoma, brain tissue undergoes rapid atrophy. This can lead to slower or, in some cases, absence of brain plasticity mechanisms [17]. Rapidly growing high-grade tumours can also produce cognitive and neurological deficits by increasing intracranial pressure or by causing brain herniation [15,26]. Low-grade tumours have been reported to cause personality or mood alterations, whereas rapidly growing tumours have been linked to major deficits in cognitive functions [12,26]. Not only the preoperative lesion volume but also the rate of tumour growth predicts NCF; indeed, patients with high-grade tumours or tumours with aggressive molecular characteristics are particularly susceptible to NCF dysfunction, regardless of lesion volume [12,27]. The relationship between tumour grade, in terms of growth rate and infiltrative characteristics, and impairment of brain networks and plasticity is crucial for rehabilitation. The tumour growth rate can considerably impact the type and severity of cognitive and neurological deficits that patients experience. Rapidly growing tumours can disrupt brain function more severely by infiltrating and displacing neural pathways, causing brain swelling, and increasing intracranial pressure. For instance, patients with low-grade gliomas often show mild impairments in language and motor skills, while those with high-grade gliomas display more severe impairments. In patients with aphasia, the rate of tumour growth may determine different pathophysiological pictures and clinical impairments, which can be explained by the progressive reduction in connections between intra- and inter-hemispheric language networks [16].

### 3.2. Therapy

#### 3.2.1. Surgical Resection

Following tumour resection, various combinations of symptoms may occur, including hemiparesis, sensorimotor deficits, visual perceptual deficits, cognitive impairment, cranial nerve deficits, dysphagia, aphasia, ataxia, and spasticity. In addition to focal neurologic deficits, patients can experience deconditioning from prolonged illness, nutritional compromise, and psychological stress. The choice of approach, the surgical resection margin, and the patient’s age are correlated with prognosis and the development of persistent and/or transient cognitive and neuromotor disorders [1,4,11,16,26,28].

In general, incomplete surgical resection, large preoperative tumour size (>4 cm), midline-crossing tumours, and adverse tissue molecular characteristics (e.g., wild-type IDH and 1p/19q deficiency) are correlated with poorer prognosis [11,26]. Age is another factor to consider when choosing surgery: complete resection appears to be associated with better progression-free survival and overall survival than limited resection in older patients with glioblastoma multiforme (GBM) [29]. Tumour location and size will influence the postoperative clinical course, with wide and deep tumours requiring dissection of the cortex and subcortical white matter [26]. In addition to functional tissue damage during resection, oedema and perioperative complications such as infarction and seizures worsen the postoperative outcome [12,27].

Patients who report new motor deficits after surgery sometimes experience them permanently but most recover within 3 to 12 months [4]. Immediately after surgery, patients may experience problems with cognitive function, including memory, executive function, and information processing speed [28]; transient aphasia may occur, especially when the superior arcuate fasciculus is involved [15]. Cognitive impairment may be mild or severe. Patients with left-sided resection showed major improvement up to 7 days post-surgery, with global cognitive improvements 3 weeks later [28]. One year after surgery, patients often show improvement in cognitive areas such as memory and learning. Those with a higher cognitive load tend to improve more. High-grade tumours or recurrence and use of antiepileptics can worsen cognitive outcomes [28]. Although surgery may initially affect HRQoL, the positive outcomes outweigh the initial negative effects. Long-term cognitive deficits that persist after surgery include impairments in visuospatial organisation, memory, information processing, and executive function.

Predictive factors for non-cognitive recovery after surgery are whole-brain radiotherapy and tumour type. These factors are associated with reduced autonomy [28]. Anxiety after surgery is quite common. The main risk factors are whole-brain radiotherapy, tumour type (especially malignant glioma or mixed histology tumours), and tumour location (mainly in the left hemisphere). Higher levels of anxiety are reported in women with left hemisphere tumours. Anxiety affects quality of life, especially when it is long-lasting (12 months after surgery) in patients with malignant glioblastoma, and when it is associated with depression. Postsurgical depression is associated with other neuropsychiatric symptoms, slow functional and physical recovery, reduced quality of life, and reduced survival. Pre-morbid depression, lower education, and female sex are risk factors for depression in this population [28].

Brain mapping [12,27] and assessment of the functional status of neuro-oncology surgery patients before and after surgery may be useful in reducing postoperative complications. Standardised assessment procedures are lacking, however. Hamer et al. suggested that functional outcome after glioma surgery should be assessed according to five indicators: neurological assessment, neurocognitive assessment, seizure onset and severity, patient wellbeing, and activities of daily living [30] (Table 1).

#### 3.2.2. Chemotherapy

Chemotherapy improves survival, but it can also be a cause of cognitive problems and fatigue [23]. More than 30% of patients with glioma experience a decline in NCF immediately after and long after chemotherapy. However, interpretation of these data is difficult due to overlapping treatments [12,26]: the negative effects on cognitive function may overlap when chemotherapy is combined with radiotherapy [26]. Temozolomide is used to treat brain tumours such as gliomas and brain metastases. It improves patients’ lives and maintains good HRQoL until the natural course of the disease is complete [12]. Factors such as type of chemotherapy, high dose, intensity, and duration are associated with increased incidence of chemotherapy-induced cognitive dysfunction (CICD) [26]. Other factors that influence susceptibility to CICD include history of head trauma, neurological disease, developmental disabilities, education, and intelligence.

Genetics can influence susceptibility to CICD, including apolipoprotein E and catechol-O-methyltransferase [26]. The most common cognitive deficits are problems with memory [16,27], processing speed, aphasia [15,16], learning [14], and executive function [26,27,28]. The effects of CICD are probably the result of multiple processes: direct neurotoxicity, myelin damage, microvascular lesions, immune-mediated processes, decreased neurogenesis and antioxidant capacity, increased oxidative stress, hormonal processes, and neurochemical imbalances [26]. The same chemotherapy drugs (e.g., temozolomide) that cross the blood–brain barrier [26] can cause cognitive, motor, and clinical damage [31]. Cognitive deficits reduce the ability to perform daily activities, maintain employment, and manage social interactions, and therefore impact the quality of life of cancer survivors [28]. To improve quality of life, the rehabilitation programme should include cognitive training, physical activity, psychological support, and symptom management [26]. Cognitive rehabilitation helps people understand their limitations. It can improve attention, verbal memory, and fatigue. Training in goal management helps to improve cognitive deficits [32,33].

#### 3.2.3. Radiotherapy

Radiotherapy is an important adjunctive treatment in the curative setting, particularly for infiltrative tumours, such as gliomas, which are seldom entirely removed by surgery alone [34]. In patients with low-grade glioma, radiotherapy may reduce progression-free survival [11]. Brain tumours can be treated with large, focused doses of radiation (stereotactic radiosurgery) as part of standard fractionated treatment or to the whole brain [35]. Fractionated doses of more than 2 Gy, higher total dose, larger brain volume irradiated, use of a dose schedule, and longer total treatment time are potential risk factors for cognitive decline after brain irradiation [35]. Compared with whole-brain radiotherapy, stereotactic radiosurgery limits the volume of healthy brain parenchyma exposed to radiation; however, adjacent structures (cranial nerves and the brainstem) are still at risk for complications [35]. Radiotherapy can cause complications that worsen the patient’s clinical condition. Radiotherapy complications can be classified according to time of onset into acute, early delayed, and late. Acute complications, occurring within days to weeks, are primarily caused by vasomotor oedema and manifest as headache, nausea, worsening of pre-existing neurological deficits, seizures, fatigue, and reversible alopecia within 2–4 months [35]. Early delayed complications, occurring within 1–6 months, result from radiation-induced demyelination and tumour or chemotherapeutic effects and manifest as neuropraxia and the drowsiness syndrome [35]. Late complications, occurring after 6 months, include cognitive decline [35,36] and radiation necrosis, leading to progressive irreversible memory loss with negative impact on HRQoL [35,37]. Radiotherapy-related cognitive decline is characterised by progressive mental slowing and impairment of attention and memory and motivation [35]. Although the quality of data is limited by the often concurrent chemotherapy, the literature reports that memory deficits are mainly associated with radiation to the hippocampus and with a low level of education [18]. Other less common radiation injuries are gait ataxia, urinary incontinence, apathy, and pyramidal and extrapyramidal signs [35].

#### 3.2.4. Drug Therapy

Antiepileptic drugs can considerably reduce psychomotor speed on perceptual tasks [11,26]. In patients with meningioma, epilepsy and the use of pharmacological antiepileptic treatment have a negative impact on HRQOL [30]. Corticosteroids administered during radiation therapy can improve symptoms of drowsiness and headaches and prevent further neurological decline [26,35].

### 3.3. Complications

#### 3.3.1. Perilesional Oedema

Perilesional oedema can occur after surgery or radiotherapy and can lead to encephalopathy, with worsening of cognitive and functioning abilities [7,26]. Postsurgical oedema can arise in the first 3 months after surgery and cause transient impairment in multiple cognitive domains (e.g., verbal memory, figural memory, working memory, executive function, psychomotor function, information processing speed, attention, language, learning, and visual construction) [16]. Oedema following acute radiation encephalopathy is related to the breakdown of the blood–brain barrier, which leads to the accumulation of fluid in the tissue (vasogenic oedema) and manifests 1–6 months after treatment, resulting in short-term memory and attention deficits and worsening of pre-existing neurological deficits [35].

#### 3.3.2. Seizures

Most patients with brain tumours (especially glioma) experience seizures [16,26]. Seizures are specific symptoms of brain tumours that result from increased intracranial pressure and direct cellular damage [12,36]. Seizure occurrence is frequent in all tumour grades; they are the most prevalent symptom during treatment and follow-up. Seizures are associated with poor cognition [18] and HRQoL [38].

#### 3.3.3. Fatigue

Fatigue can be a short-term complication of radiotherapy and an adverse effect of chemotherapy drugs such as temozolomide [32]. Over half of cancer patients experience fatigue; men report fatigue less often than women. It can impact neuropsychological assessment [26] and functionality [12] and interfere with work and cognitive performance [32]. When associated with depressed mood, it can decrease the arousal and vigilance necessary for concentration and attention [18]. It is associated with greater risk of falls, prolonged hospitalisation, and mortality, especially when combined with muscle wasting, neuropathy, and balance problems [32]. Combined rehabilitation based on physiotherapy and occupational therapy is used [7,33].

### 3.4. Outcome Measures

#### 3.4.1. Cognitive State

The most common cognitive disturbances are deficits in memory (working memory), executive function (cognitive control and flexibility, cognitive processing speed, visual searching, planning, and foresight), and general attention [1]. Anxiety and depression contribute to cognitive impairment and improve variably after intervention [18,19]. Language is related to the dominant cerebral hemisphere but it integrates an extensive network of cortico-subcortical interconnections in both hemispheres. Language is housed in association networks in comprehension functions (i.e., the uncinate fasciculus, the arcuate fasciculus, the inferior frontal-occipital fasciculus, and the superior longitudinal fasciculus). Therefore, gliomas arising in these areas are associated with a high risk of speech impairment. The supplementary motor area is related to Broca’s area, the frontostriatal tract, the frontal inclined tract, and the arcuate fasciculus [17]. According to Tariq et al., predictors of greater cognitive decline in high-grade gliomas are tumour location, tumour mass effect, and oedema, preoperative epilepsy, use of antiepileptics and corticosteroids, and advanced age at diagnosis [39]. Chemo- and radiotherapy are not good predictors of greater cognitive decline [39]. De Roeck et al. suggested that estimating long-term cognitive impairment in glioma patients would be more sensitive with the use of backward digit span, semantic fluency, the Stroop interference test, the Trail Making Test Part B (TMT B), and finger tapping [40].

#### 3.4.2. Motor Disability

Brain tumours can cause severe motor and coordination disorders and reduce independence and quality of life. Among the many factors affecting motor function are tumour location, brain oedema, perioperative morbidity, adjuvant treatment, disease progression, and psychological and cognitive aspects [4,7]. A distinction is made between perioperative and rehabilitative assessment. In patients with glioma, perioperative evaluations prioritise performance status scales, whereas rehabilitative evaluations rely on functionality scales. Nevertheless, functional outcome measures are more effective than other measures at correlating with the extent of functional independence 3 months post-surgery. Patients with impaired upper extremities may retain some autonomy but not their previous occupational roles. There is no consensus on the most appropriate functional scales. An integrated interdisciplinary approach that takes into account motor and functional deficits may provide a comprehensive overview of a patient’s health status [4].

#### 3.4.3. Response to Patient Needs and Quality of Life

Patients with brain neoplasms contend with disease-specific and general cancer symptoms that can reduce functioning and HRQoL. Specific brain tumour symptoms such as headaches, seizures, and cognitive complaints arise from increased intracranial pressure and direct cellular damage. Additional, other cancer symptoms such as fatigue, mood disturbances, nausea, and pain are common. Recurrence or progression of the disease often results in a further decline in HRQoL due to increased symptom burden, worse physical functioning, and limitations in work and social participation [12].

Large multifocal tumours, tumours located in the nondominant hemisphere, and frontal lobe tumours are associated with poorer HRQoL, pain, limited mobility, low energy, mood disturbances, sleep problems, and social isolation. A poorer functional status is noted in patients with moderate-to-severe symptoms. Noll et al. reported that affective and cognitive symptoms decrease HRQoL in long-term survivors. Activities such as walking and working are prognostic factors for HRQoL as well as for tumour progression and survival. Furthermore, the ability to work is thought to be associated with greater social participation and less financial toxicity; this aspect should be taken into account when assessing HRQoL [12].

Anxiety impacts health, quality of life, and emotional and physical functioning. It can be isolated or associated with stress and depression. Due to a lack of uniformity in assessment, its prevalence is unclear. Anxiety may occur preoperatively or postoperatively. For assessing early anxiety (within 1 month), common scales are the Hospital Anxiety and Depression Scale (HADS), the State–Trait Anxiety Inventory (STAI), and the Distress Thermometer. Women and the left brain hemisphere are more often affected. Higher levels of intermediate anxiety (1–12 months), which usually resolves within 1 year, affect the right hemisphere, while the left hemisphere persists. Higher levels of long-term anxiety (>12 months) are observed in women, people with a low level of education, and those with a history of psychiatric illness [28]. Depression, alone or associated with other neuropsychiatric symptoms, affects functional and physical recovery, quality of life, and survival; it is often associated with cortical lesions in the frontal or parietal heteromodal associative cortices, especially when it occurs early. Due to lack of uniform assessment, prevalence is unclear. When depression lasts up to 1 year or more, it can be associated with existential concerns and quality of life. Risk factors for depression after postoperative resection are: premorbid depression, lower education, and female sex. It is commonly measured with the Beck Depression Inventory and the Hamilton Anxiety–Depression Scale. Several studies found a large discrepancy between clinician-identified and patient-reported depression, suggesting the need for a more integrated and detailed assessment of mental health [28].

Patients with glioma suffer from a wide range of functional, cognitive, and psychological symptoms that dimmish their quality of life. Impairment of executive functions, whether due to tumour location or grade or treatment with radiation or chemotherapy, contributes to diminished quality of life [17]. Jzerman-Korevaar et al. found a correlation between disease stage and prevalence of certain symptoms [36]. In the diagnostic phase, cognitive deficits, convulsions, headaches, dizziness, and motor deficits are common, whereas convulsions, nausea/vomiting, cognitive deficits, fatigue, visual deficits, and anorexia are common in the treatment and the follow-up phase. A correlation exists between tumour grade and symptom prevalence: seizures are frequent in all tumour grades. Cognitive impairments are more prevalent in grade III and IV tumours, though their prevalence in grade II tumours is still considerable. The prevalence of headache is less differentiated between tumour grades. Cognitive deficits impact HRQoL (83%), with reduced return to full-time work capacity (24%), strained personal relationships (23%), and reduced independence (26%) [18,19].

Patients with meningioma may experience psychological distress if they have asymptomatic tumours or they may report a greater decline in physical health, vitality, self-care, cognition, psychomotor speed, verbal memory, working memory, and role limitations if they have symptomatic lesions that affect their HRQoL. To improve HRQoL, treatment aims to reduce specific functional, cognitive, and psychosocial symptoms [12].

An improved general state of health (better performance status) is associated with superior overall survival and progression-free survival. There is no consensus on the validation of HRQoL scales for use in glioma. Some items (motor dysfunction on the EORTC QLQ-BN20) may define quality of life, but their prognostic impact on overall survival is limited and unsupported. Other HRQoL factors that may serve as prognostic indicators for progression-free survival are motor dysfunction, nausea, vomiting, and loss of appetite. Females and younger patients have better overall and progression-free survival rates. Surgical resection and additional therapies increase overall and progression-free survival [22].

Role and cognitive function are two HRQoL variables that predict overall survival and progression-free survival. Cognitive impairment at baseline may indicate early tumour progression or act as a proxy for tumour volume. In clinical practice, HRQoL assessment monitors patient functioning and the impact of tumours and treatments on symptoms and quality of life, as well as treatment compliance [22]. A holistic approach to the patient’s health status, including motor and functional abilities and cognitive function, could move the case management perspective towards a better quality of life [4,41].

It is essential to act in the early stages of high-grade brain tumours and focus on a patient’s needs. There is also the need to provide patients and their families with consistent and well-grounded information about disease sequelae and treatment side effects; the need to understand, process, and be periodically updated on therapeutic and care choices; and the need for psychological and social support [42].

For patients with low-grade glioma, poor HRQoL impacts functioning and symptom burden. Impaired cognitive functioning and fatigue are strongly associated. While HRQoL remains stable over time, studies evaluating patients closer to diagnosis reported greater changes. Patients with low-grade glioma experience severe disturbances in cognitive, emotional, physical, and social functioning. Symptom burden is considerable due to communication deficits, fatigue, uncertainty about the future, pain, and headache-related pain. Epilepsy/seizures are the factors most strongly associated with HRQoL [38].

### 3.5. Patient-Tailored Rehabilitation

#### 3.5.1. Readjustments to Longitudinal Evolution of the Disease

The longitudinal clinical course is directly correlated with tumour evolution and subsequent chemotherapy and radiotherapy. Unlike the relatively stable course observed in stroke-related aphasia, glioma-related aphasia can, on occasion, demonstrate temporal variability. This is because the disease itself can progress or regress and treatments can affect cognitive functions. AS a result, patients may experience periods of remission alternating with phases of greater symptom severity.

Opinions diverge on the progression of cognitive deterioration over time. While some studies found that cognitive function improves following tumour resection, others reported that function does not improve or may even worsen following subsequent radiotherapy and chemotherapy and/or tumour progression [18].

Prospective studies following patients over time have reported improvements in various cognitive domains (e.g., memory, attention, and executive function) within 12 months after surgery. Patients who exhibited a higher preoperative cognitive load demonstrated more pronounced recovery. Nevertheless, decline or persistence of deficits is more frequently observed in patients who have undergone left hemisphere resection or developed a right cerebellar lesion or a malignant glioma [28]. Furthermore, whole-brain irradiation has been linked to long-term cognitive impairment, particularly in executive functioning. The estimated incidence of new-onset major depressive disorder within the initial two-week postoperative period is 4% [28].

#### 3.5.2. Neuromotor and Cognitive Treatment in the Rehabilitation Setting

Exercise improves the quality of life of cancer patients, and it is included in many guidelines for cancer survivors. A growing body of evidence supports the beneficial effects of exercise on cognition, physical well-being, and psychological well-being. In their meta-analysis, Zhao et al. reported that rehabilitation led to an improvement in autonomy both in patients with low-grade glioma and in those with high-grade glioma. Unfortunately, there are no specific guidelines for neurorehabilitation [43].

A rehabilitation plan relies on assessing and managing cognitive and physical impairment with training and reinforcement; the aim is to improve cognitive and motor function besides enhancing social participation [6]. Rehabilitation programs consist of physical activity, safe exercises, and specific cognitive treatment. Patient preferences and available resources ought to be considered when developing a feasible and sustainable exercise program [26].

Cognitive rehabilitation should be informed by neuropsychological assessment. In this context, neuropsychological screening should be performed before, during, and after brain surgery. It should also take into account comorbidities, opinions of health care professionals, and patient reports. Neuropsychological management should be carried out during periods of psychiatric and medical stability and after recovery from intensive treatment, such as radiotherapy [18]. Cognitive rehabilitation includes education, compensatory strategy training, cognitive retraining, practice, and environmental interventions to improve autonomy, self-awareness, emotional coping, acceptance, and management of cognitive impairments [12,18,19].

Bartolo et al. [7] reported that various interventions, such as general and cognitive rehabilitation, working memory training, goal management training, aerobic exercise, virtual reality training combined with computer-assisted cognitive rehabilitation, hyperbaric oxygen therapy, and semantic strategy training, have been associated with positive effects on cognition.

Strategy training focuses on compensation for NCF deficits. Several studies reported that such an approach can improve NCF impairment. On the other hand, the literature is inconclusive about the effect of cognitive retraining, a technique based on functional recovery of an impaired region. Moreover, NCF deficits seem to be improved by lifestyle interventions such as exercise [12].

Some data support cognitive rehabilitation in an acute postoperative setting, although it is unclear how much of the initial clinical cognitive gain achieved remains in the long term. The outcome of cognitive treatment in sub-acute rehabilitation is, instead, more clearly defined: many studies reported that cognitive treatment leads to an improvement within 24 days from the beginning of treatment in patients with gliomas and with metastases. Optimal timing for cognitive rehabilitation along the brain tumour disease trajectory remains elusive. Recovery is slowest in executive function and memory retrieval [18].

Various different scales evaluate the disability of neuro-oncological patients. The Functional Independence Measure (FIM) and the Karnofsky Performance Scale (KPS) are the two most widely used tools to measure functional status and degree of assistance. They investigate all components of the International Classification of Functioning, Disability and Health. The Berg Balance Scale measures static and dynamic balance skills [44]. The 6 or 10 min walk test is the most widely used scale to assess walking performance in patients with a brain tumour and chemo-radiotherapy induced impairment. The most widely used questionnaire investigating quality of life in brain tumour patients is the Functional Assessment of Cancer Therapy—Brain (FACT-BR) [44].

To assess clinical outcomes (COAs) holistically, the United States Food and Drug Administration (US FDA) has developed a set of recommendations that classify outcomes into four categories: observer-reported outcomes (based on observation by a proxy, such as a relative or caregiver), patient-reported outcomes (PROs), clinician-reported outcomes (ClinROs), and performance outcomes (PerfOs). Its acceptance is not yet common, however [4].

Correia et al. suggested Fugl-Meyer Assessment to assess PerfO, the FIM, and the World Health Organisation Quality of Life Scale (WHOQOL-Bref) to assess PRO, and the Functional Ambulation Categories to assess ClinRO [4].

#### 3.5.3. Multidisciplinary Team

A *Lancet Oncology* commission on integrating oncology and palliative care recommended moving away from a host-centred approach towards integrated, multi-professional, and patient-centred care from the moment of diagnosis onwards [45]. A multidisciplinary team can ensure care continuity and coordination, comprising acute, surgical, rehabilitative, palliative, and other interventions [7]. It includes supportive care to manage side effects and comorbidities and palliative care to relieve suffering. These services run concurrently with cancer therapies. This multidisciplinary approach is based on the biopsychosocial model, which takes into account a patient’s biological, psychological, and social needs [7]. It is designed to improve outcomes and cost savings for health care providers [46].

Multidisciplinary neurorehabilitation takes into account all aspects of well-being with the aim of improving symptoms; maximising functional independence and participation in work, leisure, and education; provide palliative care where appropriate; and optimise quality of life through a personalised rehabilitation plan [7]. In a rehabilitation setting, a multidisciplinary approach provides for optimal integration of diverse domains such as symptom control and cognitive and physical functioning through shared decision-making; a team of doctors, neuropsychologists, social workers, nurses, and mental health professionals follow patients during their hospital stay to optimise functional and cognitive recovery [7,18].

Physiatrists should be taken on board a multidisciplinary team as essential team members starting with the early stages of management [2,26]. For example, their role is to integrate the recovery of residual motor, sensory, and cognitive neurological potential, with expected satisfaction and perceived quality of life using a biopsychosocial approach in patients undergoing brain tumour surgery [4]. The multidisciplinary approach is highly valued by brain tumour patients. Based on the Patient-Reported Experience Measures (PREMs), patients undergoing radical radiotherapy reported feeling reassured, supported, and cared for by familiar staff and patient-centred care [46] (Table 2).

## 4. Discussion

People with brain tumours experience a wide range of neurological symptoms that can cause severe disability and diminish quality of life [7]. Rehabilitation responds to the complexity of patient needs according to the holistic approach of the International Classification of Functioning, Disability and Health (ICF), which classifies the health status of the disease in three levels: impairment, activity limitation, and social participation, leading to improved quality of life [7,48]. Clinical outcomes (e.g., survival, motor and cognitive function, and quality of life) reflect the effectiveness of therapy and rehabilitation [4,12,15,28]. Differently, PROMs reflect the subjective perspective of cancer and help to personalise care and improve the overall well-being of people living with a long-term condition [41,49]. Therefore, a holistic rehabilitation approach [4,41] relies on both clinical outcomes and PROMs to effectively manage the care and the rehabilitation of patients with brain tumours. The aim of the present review was to identify and evaluate predictors of functional and cognitive rehabilitation outcomes and assess their impact on quality of life in adult neuro-oncology patients. Furthermore, the review examined the impact of symptom burden, treatment side effects, and surgery on subjective and objective elements of quality of life.

### 4.1. Diagnosis (Patient Factors; Tumour Type, Site, and Grade; Genetic Factors)

In gliomas, older age and genetic variants such as carrying the apolipoprotein ϵ4 allele increase the risk of cognitive dysfunction and negatively affect rehabilitation. Dominant hemisphere or frontal involvement, growth rate, extensive surgical dissection of large and deep gliomas of the cortex and subcortical white matter also have a greater impact on cognition. In contrast, other reviews of meningiomas suggest that clinical presentation is influenced more by tumour volume, mass effect, and lateral lesion than by growth rate; in symptomatic left-sided meningiomas, clinical presentation may include language and working memory difficulties, anxiety, depression, and subjective functional impairment [49]. Conversely, supratentorial meningiomas, regardless of volume and location, may have a lesser impact on cognitive impairment and quality of life than tumours at other sites [50].

### 4.2. Surgical, Adjuvant, and Pharmacological Therapy

Treatment-related factors such as surgical, adjuvant, and pharmacological therapy also influence rehabilitation. Surgical approach, incomplete resection, molecular characteristics, and patient age influence prognosis [1,28,51,52]. Chemotherapy improves survival but can cause cognitive impairment and fatigue [36]. Radiotherapy, which is essential for invasive tumours such as gliomas, can lead to cognitive decline and minor complications. Risk is greater with fractionated doses greater than 2 Gy, higher total doses, larger irradiated brain volumes, and longer treatment times [35]. Antiepileptic drugs may impair psychomotor speed and adversely affect HRQoL, while corticosteroids during radiotherapy may improve symptoms of fatigue and headache and prevent further neurological deterioration [11,26,30,35].

The modality of neuro-oncology surgery impacts quality of life. In particularly aggressive tumours, such as GBM for example, complete resection is associated with improved progression-free survival and overall survival compared with limited resection [50]. Subtotal resection (SpTR) of selected and non-eloquent tumours may improve outcomes without substantially increasing risks compared with total resection. Outcomes are usually measured with the Karnofsky Performance Scale score, but neuropsychological function and quality of life are not fully assessed. In low-grade glioma for example, extent of resection beyond the tumour margins initially causes neuropsychological damage that normalises within 3 months, except for memory, whereas in high-grade glioma postoperative cognitive deficits develop immediately; despite some improvement in the first 2 weeks; long-term effects are often seen. Further studies are needed to better understand the benefits of SpTR for selected and non-eloquent high-grade glioma [53].

### 4.3. Complications (Perilesional Oedema, Seizures, and Fatigue)

Perilesional oedema, which can occur after surgery or radiotherapy, leads to encephalopathy and worsens cognitive and functional abilities. Post-operative oedema causes transient cognitive deficits, while acute oedema resulting from radiation encephalopathy arises 1–6 months after treatment and causes memory and attention deficits. Patients with brain tumours, particularly gliomas, are often prone to seizures due to increased intracranial pressure and direct cellular damage. This can have a negative impact on quality of life [35,36]. Further details that may be useful for rehabilitation are given in Lorimer et al., which describes a higher risk of seizures with small, slow-growing tumours, especially supratentorial and cortical gliomas [51]. Seizures are more common in secondary GBM that progresses from low-grade glioma than in primary GBM [51,54]. Early seizures are also predictive of seizures later in the disease. Seizure recurrence or worsening after a prolonged period of seizure control is highly indicative of tumour progression or recurrence. Epilepsy-associated markers are altered KCC2 activity, increased SLC7A11 expression, IDH-1 mutations, P53 overexpression, low MGMT expression, and low EGFR expression [54].

Studies have reported the efficacy of temozolomide in reducing seizure occurrence [51]. Like other antiepileptics, however, it can impair psychomotor speed [26]. Fatigue, a common side effect of radiotherapy and chemotherapy, affects more than half of all cancer patients. It impairs neuropsychological functioning and increases the risk of falls and mortality [12,18,26]. Rehabilitation with physiotherapy and occupational therapy is often given to manage fatigue [7,33]. In their study, Asher et al. reported that cancer-related fatigue is very common in patients with malignant glioma and is considered one of the most distressing symptoms. It is a strong independent prognostic factor for survival in patients with recurrent high-grade glioma, with additional prognostic value beyond performance status. There are no evidence-based guidelines for the use of pharmacological agents in the management of fatigue in patients with brain tumours. There is some evidence to support the use of stimulants for severe fatigue, but further research in this area is needed. While there is strong evidence for exercise to relieve fatigue in patients with solid tumours, no randomised trials to date have investigated this option [55].

In their study published in a Cochrane review, Day et al. found that there is currently insufficient evidence to draw reliable and generalisable conclusions about the potential benefits or harms of pharmacological (cognitive, behavioural) or non-pharmacological treatments [56].

### 4.4. Outcome Measures (Cognitive Outcome, Motor Outcome, Patient Needs, and Quality of Life)

Major factors in rehabilitation outcomes are cognitive and motor function and quality of life. Memory, executive function, and attention problems are common cognitive impairments in patients with glioma. Anxiety and depression may lead to cognitive decline [1,18,19].

Depression diminishes quality of life, making antidepressants essential for treatment; however, they can increase seizure frequency, cognitive impairment, and fatigue. In their study published in a Cochrane review, Beevers et al. reported a lack of high-quality trials on which to base guidelines [57]. An emerging area of research is brain-derived neurotrophic factor (BDNF) and its role in various brain processes: neurogenesis, differentiation, survival, synaptic plasticity, and transmission. Since it plays a role in reorganisation of the brain microenvironment, BDNF may prove useful in the treatment of memory and depression and in understanding the pathogenesis of brain tumours [58].

Language is at high risk of impairment when gliomas affect cortico-subcortical connections. Tumour location, mass effect, pre-operative epilepsy, use of antiepileptics and corticosteroids, and older age at diagnosis are predictors of greater cognitive decline [49,50]. Language and working memory appear to be more impaired in left-sided meningiomas [50]. Several studies reported the usefulness of the digit retrograde test, semantic fluency, Stroop interference test, TMT B, and finger tapping for long-term monitoring of cognitive decline, but shared standardised assessment protocols are lacking.

Brain tumours can cause severe motor and co-ordination problems and limit independence and quality of life [40]. Tumour location, cerebral oedema, treatment, disease progression, and psychological and cognitive aspects all affect motor function [39]. Patients with upper limb impairment may be able to maintain their independence, but not their previous work role. Functional outcome scales correlate better with functional independence at 3 months after surgery, although there is no consensus on the best functional scale. Besides experiencing specific symptoms (headaches, seizures, and cognitive problems), patients suffer from common cancer symptoms (fatigue, mood changes, nausea, and pain), all of which diminish quality of life. Disease progression exacerbates these symptoms and physical limitations, particularly in cases of multifocal tumours or those arising in eloquent brain areas. Anxiety and depression further impact health. Anxiety and depression seem worse in left-sided meningiomas [50]. In their study, Reinert et al. reported that the main areas of patient concern were treatment options, treatment risks, side effects, and treatment benefits [59]. Schei underlined the importance of a personalised approach to the management of glioma and the range of individual variability in cognitive response to surgical treatment. Evidence suggests that patients with high-grade glioma tend to have improved cognitive function after surgery, probably due to the reduction in tumour mass that relieves pressure on the brain, whereas cognitive function remains stable in patients with low-grade glioma. However, other factors, such as corticosteroid use, can also play a role in determining cognitive improvement, suggesting the need for personalised treatment plans that take multiple variables into account [60].

There are currently no common protocols for quality of life. An integrated approach that takes into account both physical and cognitive health is essential for better outcomes and improved survival. The US FDA has developed a set of recommendations for assessing patient outcomes divided into four categories: observer-reported, patient-reported, clinician-reported, and performance outcomes. Their acceptance is not yet widespread, however [4].

### 4.5. Patient-Tailored Rehabilitation (Longitudinal Tumour Evolution, Neuromotor and Cognitive Treatment, and the Multidisciplinary Team)

The clinical course of glioma is closely linked to tumour progression and treatment (chemo- and radiotherapy) which can affect cognition in periods of remission and symptom worsening. Evidence suggests that cognitive function tends to improve within 12 months of surgery, particularly in patients with high pre-operative cognitive reserve. More severe long-term cognitive decline is associated with left hemisphere resection, right cerebellar lesions, malignant gliomas, and whole-brain radiotherapy [12,27,28]. Based on preoperative cognitive reserve and the presence of a left hemisphere lesion, the cognitive risk should be assessed for each patient, a balance between surgical and adjuvant approaches found, and the intensity of cognitive treatment planned accordingly. The lack of robust data and longitudinal studies limits our ability to formulate accurate and tailored clinical guidelines; therefore, further research is needed to fill these gaps and improve clinical outcomes. Exercise offers many benefits to cancer patients, as it can improve quality of life and have positive effects on cognition as well as physical and psychological well-being, so much so, in fact, that Kim et al. included it in their guidelines for cancer survivors [6]. However, the guidelines lack sufficient detail to make sound recommendations for personalised rehabilitation [6,43].

Moreover, rehabilitation can improve autonomy. An effective rehabilitation plan will include assessment and management of cognitive and physical deficits, with goals for cognitive, motor, and functional improvement set out in personalised programmes [6]. Pre-, intra-, and post-operative screening and management during periods of psychiatric and medical stability are part of cognitive rehabilitation based on neuropsychological assessment [18]. Compensatory strategies and cognitive retraining, although their efficacy is inconclusive, may be enhanced by lifestyle interventions such as exercise [12]. The timing of cognitive rehabilitation is critical, with acute postoperative support and subacute rehabilitation improvement observed within 24 days. Optimal timing remains debated, however [18]. One review suggested the use of neurofeedback to improve cognitive function, depression, sleep disorders, and stress. Despite the heterogeneity of the studies, the short follow-up periods, and the low methodological quality of some of the studies, a promising area of research is to determine neurofeedback validity and efficacy [61].

Another rehabilitation approach not included in the present study is occupational therapy. In their study, Bartolo et al., suggested that occupational therapy can be useful in cancer treatment by improving activities of daily living, social participation, and quality of life. Retrospective studies and randomised trials have shown that occupational therapy in the acute setting and during active cancer treatment can improve functional and cognitive performance and reduce fatigue, making it a promising component of rehabilitation [7]. Disability is assessed using various tools (e.g., FIM, KPS, Berg Balance Scale, and walking tests), in addition to the FACT-BR quality of life questionnaire [44].

A valid tool for measuring disability is the 12-item WHODAS 2.0 scale developed by the WHO. It measures the difficulties a person may experience in different areas of daily life in the domains of cognition, mobility, self-care, interaction with others, activities of daily living, and social and community participation. The scale is particularly useful for obtaining specific domain scores [62].

The *Lancet Oncology* Commission recommended a shift from a host-centred approach to an integrated, multidisciplinary approach that puts the patient at the centre starting from diagnosis [45]. A multidisciplinary team provides continuity and coordination of care, comprising acute, surgical, rehabilitative, and palliative care [7]. This approach, based on the biopsychosocial model, takes into account a patient’s biological, psychological, and social aspects, thus improving outcomes and reducing costs [7,46]. Multidisciplinary neurorehabilitation can improve symptoms, maximise functional independence, and optimise quality of life through use of a personalised plan [7]. In the rehabilitation setting, a team of health care professionals support the patient during hospital stay for best results [7,18]. Physiatrists are essential from the early stages of treatment to integrate neurological recovery with perceived quality of life [2,4,26]. This approach is highly valued by patients who feel reassured and supported [46].

### 4.6. Patient Perspectives: PROMs/PREMs

Patient-reported outcome measures (PROMs) and patient-reported experience measures (PREMs) are of paramount importance in providing patient-centred care and assessing health care quality. PREMs help to understand a patient’s experience with the health care system, including patient information, communication with health care professionals, and quality of life. PROMs assess whether a treatment has made a difference to a patient’s health and quality of life. They are used to follow patients over time and evaluate long-term therapies and treatments. Differently, PROs identify unmet needs and improve quality of life, especially among older cancer patients in whom memory impairment, communication difficulties, and poor cooperation can affect symptom assessment. Guidelines consider patients’ preferences for treatments that improve quality of life [49].

Patients with cancer need to identify their expectations and goals for quality of life. They should then discuss their treatment options with their health care providers, weighing outcomes and identifying priorities, goals, and challenges related to quality of life. Such plan-sharing should take place many times during the therapeutic journey. In their study, Scotté identified several particularly important moments: at diagnosis, to balance the impact of disease and patient expectations; during cancer treatment, to consider side effects or the impact of cancer treatment on cancer-related symptoms; at the end of life, to balance therapeutic choices in light of life expectancy [49].

In addition to neurological and cognitive deficits, patients can experience distress related to personality changes, work problems, relationship difficulties, and uncertainty about the future. Indeed, the diagnosis itself can cause psychological distress. Patients generally have a good preoperative performance status with preserved ability to perform daily activities, work, and care for themselves, along with a reduced need for assistance [5,59,63,64].

Psychological support helps to create a favourable environment by promoting better anxiety management, effective coping strategies, and positive adaptation to the situation. It is an important part of the overall care pathway. Psychological support given in the preoperative period is helpful for patients undergoing neurosurgery. Effective coping strategies include planning, acceptance, self-distraction, and positive restructuring in the preoperative period [5] and a positive approach to the situation to facilitate adaptation [64].

In their study, Sanson-Fisher et al. analysed patient experiences and identified 14 components of cancer care in relation to the psychosocial care of cancer patients. The 14 components cover various aspects of patient care and communication: the importance of clear and timely communication in understanding diagnoses, involvement in decision making, and provision of comprehensive support throughout the cancer journey. Their checklist of guidelines identifies supportive and psychosocial care, involving clinicians, researchers, and consumers, which can be adapted to a rehabilitation-intensive neuro-oncology setting [65].

PROMs are crucial in neuro-oncology because brain tumours and their treatment affect cognitive, physical, and emotional abilities. Questionnaires can be used to measure the impact of tumours and treatment on quality of life, monitor side effects, tailor treatment, improve communication, and provide for interventions. In their study, Dirven et al. reported the need for validated survey tools for investigating specific tumour types. The 30-item European Organisation for Research and Treatment of Cancer Core Quality of Life Questionnaire (EORTC QLQ-C30), often used in conjunction with the EORTC QLQ Brain Neoplasm (BN20) and the FACT-BR, which are most commonly used in interventional and observational studies. Many tools have not been specifically designed or validated for all types of brain tumours, however, and require further research into their content validity and reliability and consistent measurement properties [30,66].

The most commonly reported symptoms in the course of glioma are seizures, cognitive impairment, somnolence, dysphagia, headache, confusion, aphasia, motor deficits, fatigue, and dyspnoea; their frequency varies greatly in different stages of the disease [36].

In their study, Schiavolin et al. reported that the factors associated with poorer PROMs are male sex, WHO grade 4 histopathology, greater procedural complexity, and a higher complications rate. Cranial neurosurgery does not appear to affect long-term psychological well-being, even when complications are present [67].

Other variables that may influence psychological well-being are treatments and therapies (e.g., radiotherapy and chemotherapy), tumour histological grade, psychological and social impact of the disease, and concerns about tumour recurrence [41]. Furthermore, emotional and cognitive impairment directly affect quality of life and length of survival [17].

Antiepileptics have a negative impact on HRQoL regardless of seizure frequency. In patients with meningioma and preoperative seizures, surgical resection is often effective in improving seizure control and reducing antiepileptic drug use. Treatment of meningiomas can often preserve or even improve function and HRQoL. Most patients undergo surgical resection and/or radiotherapy. The degree of improvement in HRQoL varies with pre-treatment symptom burden.

A common pattern is an initial decrease in HRQoL after radiotherapy, followed by a period of normalisation or improvement from baseline over long-term follow-up [52]. The most common low-grade treatment toxicities are local alopecia and radiodermatitis [52]. The symptoms may worsen the patient’s experience and impact their discomfort and psychological state. PROMs collect data on how caregivers cope with stress, their mental health, their ability to maintain their own well-being while caring, and any special needs they may have. In their review, Heinsch reported on the impact of the patient’s condition on a carer’s psychological well-being. They found that psychological well-being is linked to the progression of a loved one’s illness and that the organisational network is inadequate to support them [68].

By incorporating caregiver-focused patient-reported outcome measures (PROMs), health care providers can more effectively support both patients and their families. This approach ensures that interventions are designed to help patients and reduce caregiver burden. Such an integrated approach promotes a holistic model of care that addresses the unique challenges faced by caregivers (Table 3).

Table 4 presents improvement as suggested by PREMs and PROMs.

PROMs, PREMs, and predictive outcome measures of the diagnostic and therapeutic care pathway (PDTA in Italy) are structured around a patient, ensuring a patient-centred approach that treats the disease and improves quality of life. This approach helps health care organisations to be more responsive and to focus on continuous performance improvement. The definition of integrated care pathways for cancer patients is important for developing interdisciplinary collaboration (neuro-oncologists, neurosurgeons, neuroradiologists, and physiotherapists) and improving care pathway quality. Clinical pathways should define multidisciplinary care plans that translate guidelines into practical intervention. They consider diverse sub-populations, adapt to local contexts, and predict challenges through accurate algorithms [10].

A multidisciplinary team and a multi-professional approach to patient care offers the best chance to manage all aspects of a patient’s health and improve treatment efficacy on overall health and quality of life [44,69].

Multidisciplinary integration in the treatment of brain tumours plays a fundamental role in personalised medicine, allowing treatment to be tailored to the specific characteristics of the tumour. By integrating the expertise of each team member, more informed surgical and therapeutic decisions can be made, increasing treatment efficacy and improving patients’ quality of life. For example, molecular analysis allows the identification of genetic mutations and biomarkers that guide surgical and therapeutic decisions, both at the level of adjuvant therapies and in identifying the risk of complications, such as the risk of epilepsy, and the expected response to rehabilitation [12,18,24,25,26]; advanced imaging techniques and cognitive assessments help neurosurgeons to plan safe resections that preserve critical brain functions, and rehabilitation specialists to plan the expected recovery, both immediately after surgery and in the long term [12,26,27,28].

Having different specialists on the team reduces the risk of neglecting patient needs. An integrated approach that targets quality of life goals and clinical needs can achieve this even better. PREMs show that patients feel reassured and supported by multidisciplinary treatment and personalised care from familiar staff. Members of a multidisciplinary team vary widely by unit and country. As neurorehabilitation is an integral part of brain tumour management, we believe physiatrists should be part of the care team [46].

### 4.7. Rehabilitation

Rehabilitation is essential for patients with benign and malignant brain tumours; therefore, it should not be left out of a treatment plan. Patients with benign tumours tend to have greater functional independence than those with malignant tumours [70]. As patient survival increases, so does the need for personalised rehabilitation to improve functional recovery, social participation, and quality of life [69]. A holistic approach to rehabilitation, which takes into account clinical outcomes and patient-reported outcome measures (PROMs), is crucial [4,41]. This approach, aligned with the International Classification of Functioning, Disability and Health (ICF), addresses the health status of disease on three levels—impairment, activity limitation, and social participation—and improves quality of life [7]. Patient satisfaction depends on factors related to health care professionals and the patient’s own characteristics [10]. Planning and sharing rehabilitation plans with patients and their carers can increase rehabilitation effectiveness and adherence to therapy, improve multidisciplinary management, and address emotional aspects such as shock, anxiety, and depression [2]. Rehabilitative hospitalisation should be reasonable and necessary, provided by a coordinated multidisciplinary team [18,44,71] and tailored to the patient’s needs and outcome measures, with constant physiotherapy [63], intensive care (at least 3 h/day, at least 5 days/week), and a rehabilitation doctor 6 days a week to assess and treat medical and functional outcomes. It should include active participation and meaningful benefits through a tailored intensive rehabilitation program [7,71,72].

Exercise and rehabilitation can reduce the side effects of invasive and pharmacological therapies, reduce fatigue and muscle wasting, increase physical endurance, and enhance participation in daily activities and health-related quality of life [44,72]. Neuropsychiatric outcomes (e.g., cognition, depression, and anxiety) are common post-surgery and should be assessed with standardised protocols to monitor their impact on quality of life [28]. In order to achieve positive long-term treatment outcomes, caregivers need to be involved in the rehabilitation plan [70].

Moreover, this overview included narrative reviews and studies involving small samples or characterised by temporal validity limitations; these aspects may limit data interpretation; nevertheless, they encourage better design of high-quality studies to provide the most comprehensive care possible in clinical practice to patients with brain tumours.

## 5. Conclusions

To study the impact of symptom burden and treatment side effects on subjective and objective elements of quality of life in patients with primary CNS tumours, a holistic approach to rehabilitation will take into account clinical outcomes and PROMs [4,41]. Robust guidelines on predictive outcome criteria and the patient’s point of view are needed. Care pathway management should include quality-of-life scales and cognitive scales. Future interventions to improve PREMs and PROMs should include personalisation of questionnaires, adapting them to the specific work and clinical needs of patients in simple language; active patient engagement in selecting questions relevant to their experiences; use of digital technologies such as apps and digital platforms for data collection; continuous monitoring and systems to monitor outcomes over time; feedback based on PROM and PREM data provided to clinicians; and involvement of family members, with consideration of family member experiences in designing the questionnaires.

Multidisciplinary teams can ensure better outcomes and greater cost-effectiveness. Early evaluation by a rehabilitation physician is essential to assess neurological changes after brain tumour removal in terms of functionality, limitations, work capacity, and social interactions. Protocols and guidelines for motor, cognitive, and occupational recovery are essential tools in rehabilitation.

## Figures and Tables

**Table 1:** Predictors of surgical outcome. Observational risk factors and rehabilitation implications for each variable.

Variable Type		Risk Factor	Effects on Rehabilitation	Ref.
Surgery	Incomplete Resection	Large tumours (>4 cm), midline-crossing tumours, unfavourable (IDH wild-type, 1p/19q deficiency)	Poorer prognosis, slower functional recovery	[8,23]
	Patient Age	Older patients with GBM	Complete resection associated with better survival and rehabilitation than limited resection	[26]
	Tumour Location and Size	Large, deep tumours	Require dissection of the cortex and subcortical white matter, with negative effect on postoperative course	[23]
Complications	Oedema and Perioperative Complications	Oedema, infarction, seizures	Worse postoperative outcome and complicated rehabilitation	[9,24]
Outcome	Post-operative Motor Deficits	New motor deficits after surgery	Most patients recover within 3–12 months, but some may have permanent disabilities	[4]
	Post-operative Cognitive Function	Immediate problems with memory, executive function, information processing speed	Global improvement up to 3 weeks post-surgery, cognitive improvement at 1 year	[26]
HRQoL	Post-operative Anxiety and Depression	Whole-brain radiotherapy, tumour type, tumour location (left hemisphere), female sex	Anxiety and depression have a negative effect on quality of life and rehabilitation, with long-term effects	[26]

**Table 2:** Outcome Predictors. The variables in rehabilitation are briefly reported; details are given in the text.

		Medical Description	Citations
Diagnosis (patient characteristics; tumour type, site, grade; genetic factors)		Patient age, tumour site, genetic and molecular characteristics, and Karnofsky Performance Scale (KPS) scores (Weller 2022) are determining factors for functional impairment and prognosis recognised by the World Health Organization (WHO). Clinical patterns are influenced by tumour site and its impact on the functional neurocognitive neuronal network. In cases where eloquent brain areas, particularly subcortical tracts, are affected, there is a negative correlation with cognitive, functional, and behavioural recovery. The speed of tumour growth affects the clinic as a function of anatomical damage. Infiltration, interruption, and displacement of fibrous tracts; perilesional oedema; and surrounding neuronal sparing create specific clinical patterns. Polymorphism in the ApoE allele correlates with aphasia. In patients undergoing adjuvant therapy, the presence of at least one ApoE ε4 allele is associated with deficits in verbal memory and executive functions. There is no consensus on the use of ApoE ε4 testing as a neurocognitive predictive criterion for post-surgical outcomes.	[11,12,13,14,15,16,17,18,19,20,21,22,23,24,25,26,27]
Therapy	Surgical Resection	Type of approach, surgical resection margin, and patient age may be correlated with prognosis and cognitive and neuromotor disorders, which, in some cases, are transient.	[1,4,11,12,15,16,26,27,28,30]
	Chemotherapy	Many side effects diminishing quality of life observed after chemotherapy: transient aphasia, learning, memory, processing speed, executive functions, and fatigue.	[11,12,15,17,18,26,27,31,36]
	Radiotherapy	The dose-limiting morbidity of CNS radiotherapy is late radiation injury manifesting as focal injury or diffuse insult. The impact on quality of life and cognitive deficits remains debated.	[11,12,18,27,31,34,35,37,47]
	Antiepileptic Therapy	Antiepileptics can slow psychomotor speed on perceptual tasks.	[11,26,30]
	Corticosteroids	Corticosteroids during radiation therapy can improve drowsiness and headaches and prevent further neurological decline.	[26,35]
Complications	Perilesional Oedema	Perilesional oedema can arise after surgery or radiotherapy and lead to acute or delayed encephalopathy, frequently causing or worsening cognitive impairment.	[16,26,35]
	Seizures	Seizures are the most common symptom during treatment and follow-up in all tumour grades.	[12,16,26,36]
	Fatigue	Fatigue is a frequent side effect of chemotherapy; fatigue affects prognosis; men report fatigue less often than women.	[12,18,26,33]
Outcome Measures	Cognitive State	Anxiety and depression contribute to cognitive impairment. Cognition should be assessed before, during, and after an intervention to quantify damage, allow adequate surgical resection, and initiate personalised neuropsychological treatment.	[1,17,18,19,39,40]
	Motor Dysfunction	Motor dysfunction in patients with primary CNS tumours may be multifactorial, related to direct effects of tumour site or brain oedema or adjuvant therapy.	[4]
	Response to Patient Needs and Quality of Life	Anxiety and depression diminish quality of life. Patients with glioma present with a wide range of symptoms that reduce their quality of life. Anxiety and depression reduce quality of life. A holistic approach to health status, including motor and cognitive function, may improve case management, leading to improved quality of life Early intervention is crucial for treating high-grade brain tumours, with a focus on patient needs. Impaired executive functions in patients with glioma impact their quality of life. Patients with asymptomatic meningioma may experience reactive psychological distress, whereas those with symptomatic meningioma report greater deterioration in physical health, vitality, self-management, cognitive function, psychomotor speed, verbal memory, working memory, and role limitations.	[4,12,17,18,19,22,28,36,38,41,42]
Patient-tailored Rehabilitation	Readjustment to Longitudinal Evolution of Disease	The longitudinal course of brain cancer is influenced by tumour progression and the effects of chemotherapy and radiotherapy. Glioma-related aphasia often fluctuates, reflecting changes in the disease, with alternating periods of improvement and worsening. Cognitive recovery varies; some patients improve after resection, while others deteriorate further after additional treatments. Improvements in cognitive functions are generally observed within 1 year.	[18,28]
	Neuromotor and Cognitive Treatment in Rehabilitation Settings	A growing body of evidence supports the beneficial effects of exercise on cognition, physical, and psychological well-being. There are no specific guidelines for neurorehabilitation. Predictive assessment scales are the Functional Independence Measure (FIM), the Karnofsky Performance Scale, the Berg Balance Scale, the 6 or 10-min walk test, the FACT questionnaire (including FACT-B).	[6,12,18,19,26,44]
	Multidisciplinary Team	Patients with glioma experience positive outcomes when treated by a multidisciplinary team. A collaborative approach improves results, while reducing costs and ensures effective management of symptoms and cognitive function. A patient-centred multidisciplinary team should include physicians, neuropsychologists, social workers, nurses, and mental health experts. Early evaluation by a rehabilitation physician is crucial for assessing neurological changes such as motor, sensory, and cognitive deterioration after brain tumour resection, while considering patient satisfaction with quality of life in terms of functionality, impairment, work capacity, and social interaction.	[2,4,6,18,26,43,46]

**Table 3:** PROMs and quality-of-life effects.

Clinical Symptoms	Seizures, cognitive impairment, somnolence, dysphagia, headache, confusion, aphasia, motor deficits, fatigue, and dyspnoea Are symptoms reported by glioma patients. The prevalence varies across disease stages.
Therapy	Cranial neurosurgery does not substantially impact long-term psychological well-being, even when complications are present. Other factors that may negatively influence psychological well-being are additional treatments (e.g., radiotherapy and chemotherapy), histological tumour grade, psychological and social impact of the disease, and concern about tumour recurrence. Antiepileptics have a negative impact on HRQoL regardless of seizure frequency. Surgical resection of meningiomas often improves seizure control and reduces antiepileptic drug use.
Complications	A higher rate of surgical complications correlates with poorer patient-reported outcomes. Local alopecia (hair loss) and radiodermatitis can increase discomfort and reduce psychological well-being.
Emotional State	Emotional state and cognitive impairment directly affect quality of life and survival time. Concern about tumour recurrences has a negative impact on well-being. Family caregivers play a central role in providing practical and emotional support.

**Table 4:** Improvements as suggested by PREMs and PROMs.

		How to Improve PROMs/Quality of Life	How to Improve PREMs
Diagnosis	Tumour type	Inform patients clearly about their health conditions, the natural history of the disease and therapeutic options; plan a genetic, neuroradiological, and clinical diagnostic path; identify a reference tutor for the diagnosis; provide contact tutor details; provide a diagnosis within a reasonable timeframe.	Use a patient-centred communication and interaction style; disclose diagnosis and prognosis when desired, with support persons present. Provide prognostic and outcome information before, during, and after treatment. Share the care pathway. Provide adequate psychological support, especially in cases of highly malignant tumours; establish a working relationship with caregivers and train them how to manage disability.
	Tumour site		
	Tumour growth rate		
	Genetics		
Therapy	Surgical resection	Inform patients clearly about the risks and benefits of surgical treatment; test function and cognition before and after surgery.	Adopt a patient-centred approach for communication and interaction. Ensure that doctors are available to inform and initiate the care pathway and clinical/care management. Share the care pathway with the patient (and support persons). Manage symptoms and side effects. Ensure long-term continuity of care after treatment. Provide psychological support to help patients identify coping strategies and define their experiences. Encourage conversations among cancer patients to share their own experiences. Develop a tailored rehabilitation program.
	Chemotherapy	Provide for medical support to inform and start the treatment path and the clinical/welfare management.	
	Radiotherapy	Ensure the presence of medical support to inform and start the treatment path and the clinical/welfare management.	
	Antiepileptic therapy	Inform the patient about the potential harm caused by antiepileptics.	
	Corticosteroids	Inform the patient about the benefits and possible risks of corticosteroid use.	
Complications	Perilesional oedema	Inform the patient about correlations between deficits and perilesional oedema and the longitudinal evolution of the problem.	
	Seizures	Patient information.	
	Fatigue	Patient information.	
Outcome measure	Cognitive state	Neuropsychological evaluation before, during, and after surgery and adjuvant therapy; ensure adequate treatment intensity according to the tailored PRI. Inform the patient.	Ensure a patient-centred communication and interaction style. Develop a rehabilitation program tailored to each patient, considering outcome measures and patient needs. Share the rehabilitation plan with patients and their caregivers. Conduct cognitive assessment using specific neuro-oncological test batteries. Provide clear rehabilitation prognostic information. Address and adequately inform about complications that may negatively impact the outcome and quality of life. Ensure the involvement of a referee physiatrist and a neuropsychologist in the treatment path. Encourage conversations among cancer patients to share their experiences related to the rehabilitation plan.
	Motor dysfunction	Ensure adequate treatment intensity based on the tailored PRI. Inform the patient.	
	Response to patient needs and quality of life	Assessment of patient needs; create a rehabilitation strategy flexible over time based on diagnosis and life prognosis; sharing of achievable goals by the team and the patient (and caregivers); time planning and methods for achieving objectives; monitoring achievement of objectives expected by the patient; patient-reported quality monitoring; psychological support; psychiatric support.	
Patient-tailored rehabilitation	Longitudinal evolution	Ensure clinical/functional monitoring by a multidisciplinary team.	Ensure effective rehabilitation through periodic physical assessments and meetings, creating a personalised and ongoing approach over time. Adopt a patient-centred approach with age- and outcome-specific treatment pathways to enhance quality of life. Identify physical, psychological, social, and neuropsychological symptoms and side effects across the rehabilitation and disease continuum to improve communication and decision-making. Recognise patient experiences and unexpressed needs. Identify and redefine what is possible and important. Engage support persons (if shared and possible) and maintain ongoing communication throughout rehabilitation. Create a multidisciplinary team to identify, share, and coordinate cognitive, functional, neuropsychiatric, neurosurgical, neuroradiological, neurooncological, geriatric, psychological, physical, neuropsychological, and speech therapy needs. Conduct regular multidisciplinary meetings focusing on the patient’s informational, communication, psychological, social, spiritual, and rehabilitation needs.
	Neuromotor and cognitive treatment in rehabilitation	Plan tailor-made rehabilitation according to patient needs; identify caregivers, ensure an evidence-based rehabilitation approach. Use a team approach that includes occupational therapy, physical therapy, speech therapy, and social work.	
	Presence of a multidisciplinary team	An integrated multidisciplinary and patient-centred approach can improve management quality, especially for the elderly with GBM; conduct a geriatric evaluation in elderly patients with different comorbidities, physiological and cognitive functioning, and performance status when screening and during care management; assess motor, sensory, and cognitive neurological deterioration considering patient satisfaction with perceived quality of life; assess neuropsychiatric outcomes, with standardised preoperative and postoperative protocols affecting quality of life. Shift the focus of care from a patient-centred to a family-centred perspective when the prognosis suddenly worsens.	
